# Initial Survival and Development of Planted European Beech (*Fagus sylvatica* L.) and Small-Leaved Lime (*Tilia cordata* Mill.) Seedlings Competing with Black Cherry (*Prunus serotina* Ehrh.)

**DOI:** 10.3390/plants9060677

**Published:** 2020-05-27

**Authors:** Sarah L. Hasstedt, Peter Annighöfer

**Affiliations:** 1 Department of Silviculture and Forest Ecology of the Temperate Zones, Georg August University of Göttingen, Büsgenweg 1, 37077 Göttingen, Germany; 2 Forest and Agroforest Systems, Technical University of Munich (TUM), Hans-Carl-von-Carlowitz-Platz 2, 85354 Freising, Germany; peter.annighoefer@tum.de

**Keywords:** invasive species, black cherry management, light availability, seedling mortality, biomass allocation, specific leaf area

## Abstract

Black cherry (*Prunus serotina* Ehrh.) is considered one of the most invasive tree species in central Europe and causes problems for both nature conservation and silviculture. Besides mechanical control treatments, a suggested control method to prevent its ongoing spread is to underplant shade-tolerant native tree species. Therefore, we combined two mechanical treatments, with underplanting of European beech (*Fagus sylvatica* L.) or small-leaved lime (*Tilia cordata* Mill.) on fenced and unfenced plots. After the first growing season, survival rates were evaluated, and selected seedlings were destructively harvested to analyze their growth performance and leaf morphology in association with the different light regimes resulting from mechanical treatments Survival rates for both seedlings were very high (>95%). Survival rates were higher on fenced plots than on unfenced plots, most likely as result of browsing. The mortality of *F. sylvatica* decreased with increasing light availability on fenced plots. The mortality of *T. cordata* did not change along the light gradient. After one vegetation period no differences with respect to biomass allocation could be detected along the light gradient. However, the specific leaf areas of both species responded similarly, decreasing with increasing light availability. In summary, both species were able to establish and survive in the dense *P. serotina* understory and might have the potential to outcompete the invasive alien species in the long run.

## 1. Introduction

Biotic invaders can inflict enormous environmental damage to ecosystems by altering the integrity of their fundamental properties [1]. Invasive plant species affect competition for resources like water or nutrients, and can change the invaded ecosystems’ components such as increased biomass production or nitrogen availability and litter production with higher decomposition rates for instance [2]. They are globally recognized as major threats to biodiversity, nature conservation, and ecosystem stability in native forests [1,3]. The urgent question, when dealing with any kind of invasive species, is how to handle or control them after their presence is discovered to avoid or minimize possible damage to the ecosystem.

The deciduous tree species black cherry (*Prunus serotina* Ehrh.), native to North America [4], is the largest cherry in its native range [5] and is a valuable furniture wood in the United States [6]. *P. serotina* grows well on a wide variety of soils but grows best on the Allegheny Plateau in the eastern United States of America [5]. With the aim of producing timber of high commercial value [6], the species was introduced to Europe in the early 17th century through France [7,8]. Since the late 18th century, the species has been planted in Germany [9] for several purposes [10]. Aside from producing high quality timber, foresters hoped to improve soil qualities of degraded sites through the species decomposable litter and to reduce the susceptibility of existing pine stands to fire. In addition, *P. serotina* was planted as fire breaker in highly valuable stands. However, on nutrient-poor sandy soils, the growth forms were disappointing [9] because instead of growing into valuable high-quality timber, stems were often crooked with crotches [11,12]. In many cases, *P. serotina* that was planted for fire prevention [9], spread uncontrollably and was hence considered a “wood pest” across western Europe [13]. In the 1950s, Belgium and the Netherlands stopped *P. serotina* plantings, while German foresters continued planting the species until the 1980s [9].

Currently, *P. serotina* grows from the north of France to Poland, Hungary, and Romania as well as from Denmark to Italy [14] and is one of the most invasive plants in Europe [15]. In Germany, high abundances of *P. serotina* are mainly found in Scots pine (*Pinus sylvestris* L.) forest stands in Schleswig-Holstein [16], Brandenburg, and Berlin [9], the northern German plain, Rhineland-Palatinate, and also on sandy soils in southern Germany in Bavaria and Baden-Württemberg (Federal Agency for Nature Conservation. Center for German Phytodiversity). The high abundance of this non-native species in managed forests is considered to be a result of long-term planting activities rather than of natural regeneration and dispersal [12]. Once established, efforts to manage and control large affected areas have turned out to be time-consuming, labor and cost intensive, and often ineffective [16,17,18,19]. Complete removal of *P. serotina* from forest ecosystems is considered to be nearly impossible [10]. Felled *P. serotina* trees quickly produce stump and root suckers [5,19,20]. Girdling the trees seems to be more effective, but is very labor intensive [21]. Under poor light conditions, only minimal radial growth (<0.06 mm) is observed in *P. serotina* seedlings [20,22]. However, when light availability suddenly increases due to disturbance-induced canopy gaps (e.g., from thinning, clearing, storm damage), seedlings quickly grow into the new gaps [20]. Due to their growth characteristics, pine forests with relatively low canopy densities are especially susceptible to be invaded by *P. serotina*. An alternative to mechanical treatments might be to underplant invaded stands with shade-tolerant species to shade-out *P. serotina* over time [23,24,25]. This control method for *P. serotina* has been applied and tested as a management option for invaded forests in Germany in only a few studies to date [26,27,28]. A long-term study with underplanted European beech (*Fagus sylvatica* L.) and Douglas fir (*Pseudotsuga menziesii* (Mirbel) Franco) [28], has shown the competitive strength of *F. sylvatica* to survive and grow under *P. serotina* growing in birch stands. Case studies [26,27,28] showed by high survival that small-leaved lime (*Tilia cordata*, Mill.) as well as other native shade tolerant tree species such as European hornbeam (*Carpinus betulus* L.) could also be promising for this purpose. To our knowledge, no study exists that combines underplanting native species with mechanical treatments applied to *P. serotina*.

We planted (fenced and unfenced) 2-year-old, bare-rooted seedlings of *F. sylvatica* and *T. cordata* in a managed pine-forest with a pronounced *P. serotina* understory (between 8 and 15 m in height) in combination with two common mechanical treatments in forest management (felling and girdling). The treatments resulted in different levels of light availability in the understory. Seedlings were monitored during the vegetation period and a representative number was harvested at the end of the vegetation period to evaluate biomass allocation and growth patterns within the plants as reaction to the light regimes. These helps identify appropriate conditions to underplant the seedlings and to maximize their competitive ability against *P. serotina*. The hope is that the native shade tolerant species can at least keep up with the invasive *P. serotina* and shade-out the species in the long run. 

Tree growth, driven by photosynthesis, is defined as the increase in total plant dry mass. This mass is allocated in different tree compartments (leaves, branches, stem, roots) [29,30]. This distribution of biomass across the plant provides insight into the plants’ growth performance and concomitantly their access to limiting resources such as nutrients, water, and light [29]. The present study focused on biomass allocation of underplanted tree-seedlings under different light conditions in order to evaluate their growth performance.

The aims of this survey were (a) to study whether the chosen underplanted tree species survive in the dense *P. serotina* understory, (b) to relate biomass allocation patterns in the small seedlings to light availability, and (c) to relate specific leaf area to the light available as a result of previously conducted mechanical control-methods applied to the invasive species.

## 2. Results

### 2.1. Mortality

#### 2.1.1. Seedling Mortality Inside and Outside the Fences

**Figure 1:**
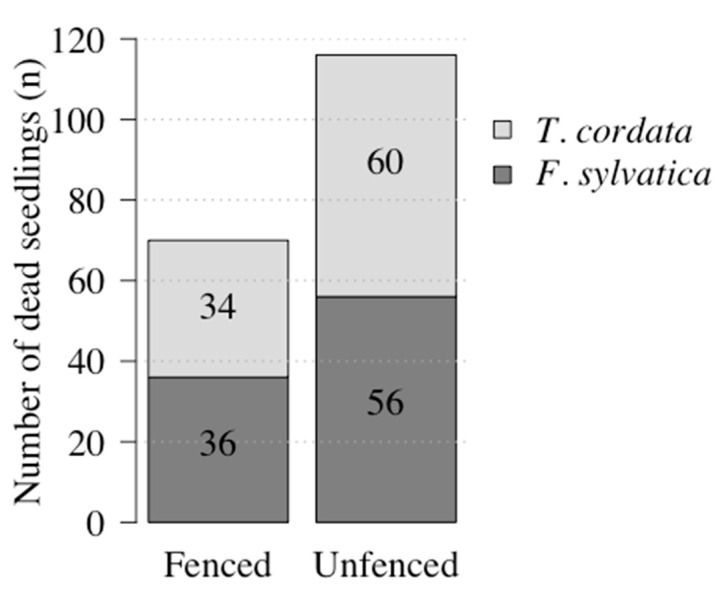
Absolute number of dead *Fagus sylvatica* and *Tilia cordata* seedlings on fenced and unfenced plots after the growing season 2017 in the whole experiment.

After the first growing season 2017, the overall mortality of both species was negligible (Table S1 in supplementary material contains a complete dataset on dead seedlings). Of the 2108 *F. sylvatica* seedlings, 92 died and of the 2062 *T. cordata* seedlings, 94 died, which equaled an overall mortality of 4.4% and 4.6% and indicated no differences in mortality between species (Chi-squared; *p* = 0.819). For both species, the number of dead seedlings was higher on unfenced plots compared to the fenced plots (*F. sylvatica*: *p* = 0.038 and *T. cordata*: *p* = 0.005). No difference between the two species could be detected within (*p* = 0.946) or outside the fences (*p* = 0.643) (Figure 1).

#### 2.1.2. Mortality along light gradient

**Figure 2:**
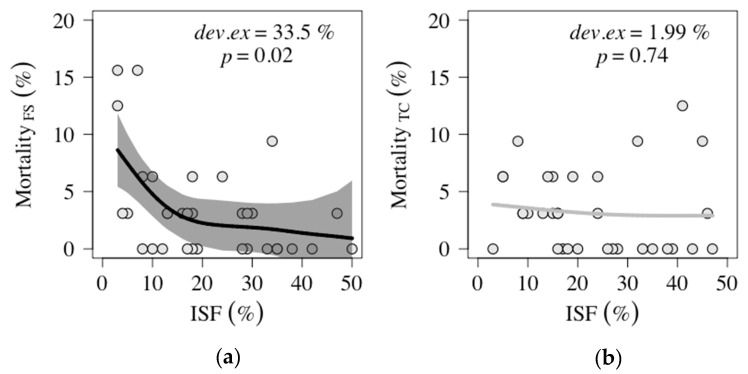
Mortality of (**a**) *F. sylvatica* seedlings (FS) and (**b**) *T. cordata* seedlings (TC) on fenced plots (*n* = 33) in association with light, measured as indirect site factor (ISF). Significant trends (*p* < 0.05) in association with light are shown as a black line, non-significant trends as a grey line. Shaded grey area shows the 95% confidence interval. Both explained deviance and *p*-values were extracted from the generalized additive model (GAM)-models.

On average, one seedling died on each plot over the entire field experiment. Note that a single dead seedling represented a mortality of 3.13% (Table 1). The fenced and unfenced plots did not differ in light availability (*p* = 0.294). Thus, the further analysis of mortality and growth performance (seedlings’ biomass, compartment mass fraction analysis, and specific leaf area) was restricted to the fenced sites, to exclude an effect of browsing. The correlation between mortality and light availability was only significant for *F. sylvatica* seedlings, with mortality decreasing with increasing light availability (Figure 2a). No correlation between mortality and the light availability (ISF, indirect site factor, Table S3 in supplementary material contains all abbreviations and definitions used in this manuscript) was detected for *T. cordata* seedlings and the calculated GAM (generalized additive model) was also not significant (Figure 2b).

### 2.2. Seedling Biomass 

#### 2.2.1. Biomass before and after Growing Season

Due to browsing damage on a side shoot, one *F. sylvatica* seedling had to be excluded in this analysis. The seedlings’ dry mass, calculated as total dry mass (TDM), aboveground biomass (AGB), and root mass (RM), were compared before and after the first growing season for both tree species. Changes could not be detected, neither in *F. sylvatica* (Kruskal-Wallis-Test: 0.07 < *p* < 0.11) nor in *T. cordata* (0.17 < *p* < 0.24) (Table 2).

#### 2.2.2. Response in Mass Fractions along the Light Gradient

The three tested stands (b2, c2, c3) did not reveal a significant difference on the mass fractions (MF) of leaves (LMF), branches (BMF), stem (SMF), and roots (RMF) neither for *F. sylvatica* (0.14 < *p* < 0.70) nor *T. cordata* (*p* = 0.06 < *p* < 0.65). The treatment of *P. serotina* itself also showed no significant difference on the compartment mass fractions for *F. sylvatica* (0.82 < *p* < 0.94) or *T. cordata* (0.26 < *p* < 0.55). Table S2 (supplementary material) contains the complete seedlings dataset on mass fractions.

**Figure 3:**
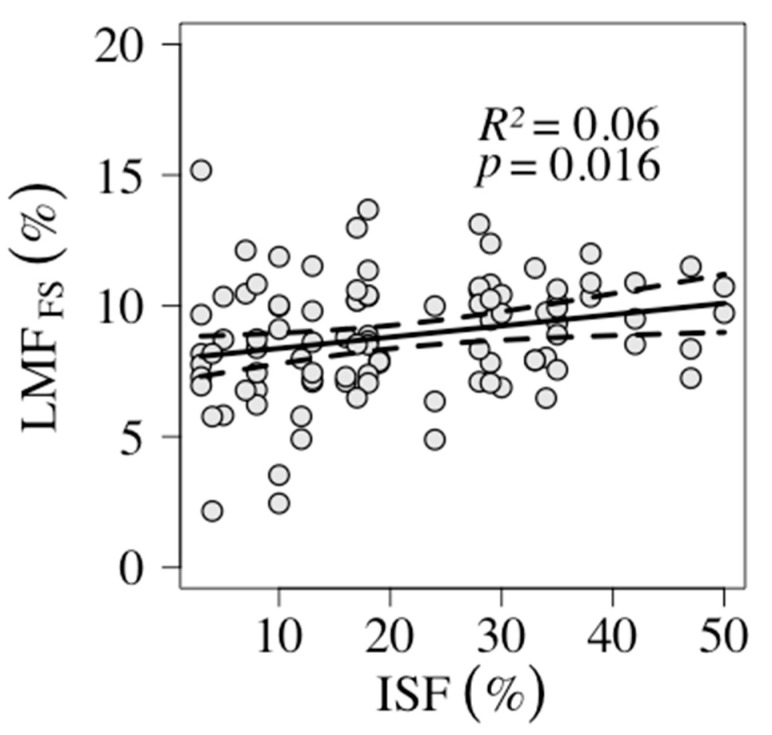
Leaf mass fraction (LMF) of *F. sylvatica* seedlings (FS) from fenced plots in association with indirect site factor (ISF). The sample-size was *n* = 98. Black solid line shows the trend and the shaded grey area shows the 95% confidence interval. *R*^2^ and the *p*-value were extracted from the GLM (generalized linear model).

The LMF of *F. sylvatica* seedlings was the only mass fraction that showed a significant trend along the light gradient (Table 3). The LMF increased by 0.04% with 1% light availability, measured as ISF (Figure 3). The three other mass fractions in *F. sylvatica* (BMF, SMF, RMF) and also AGB did not respond along the gradient (0.127 < *p* < 0.310) (Table 3). AGB and none of the mass fractions of *T. cordata* showed a significant trend along the light gradient (0.127 < *p* < 0.841) (Table 3).

### 2.3. Specific Leaf Area

Three harvested *F. sylvatica* seedlings with only dried-out leaves had to be excluded in this analysis. The mean leaf area (LA) did not respond along the light gradient (*F. sylvatica p =* 0.936; *T. cordata* *p =* 0.658). The specific leaf area (SLA) in *F. sylvatica* leaves ranged from 170.7 to 307.0 cm^2^g^−1^, which was on average less than for *T. cordata* leaves ranging from 190.6 to 444.8 cm^2^g^−1^. SLA was on average 25.3% greater in leaves of *T. cordata* (*p* < 0.001) (Table 4).

SLA was negatively correlated with light in both tree species, as SLA decreased with an increase in light (both species: *p* < 0.001) (Table 3). A greater proportion of the variance in SLA was explained by light in leaves of *T. cordata* (*R*^2^ = 0.21) in comparison to leaves of *F. sylvatica* (*R*^2^ = 0.13) (Table 3).

**Figure 4:**
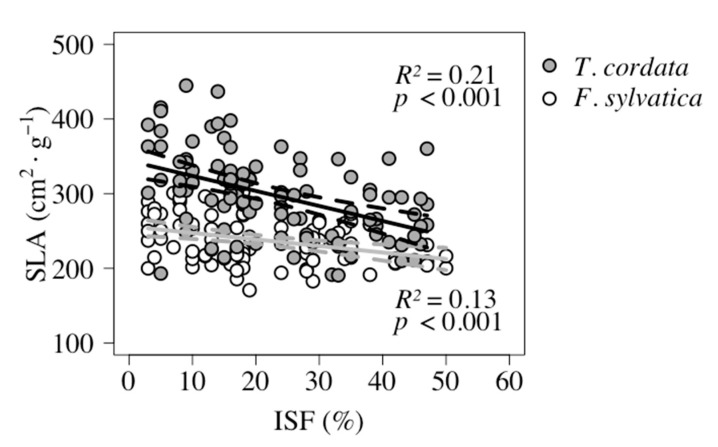
Specific leaf area (SLA) in association with light, measured as indirect site factor (ISF). Sample size for *F. sylvatica* was *n* = 96 and for *T. cordata n* = 99. The trends were significant for both tree species (*p* < 0.001) and are shown as solid lines (black shows the trend for *T. cordata* and grey shows the trend for *F. sylvatica*). Dashed lines show the 95% confidence intervals.

A comparison of the slopes in both models revealed significant differences (*p =* 0.02) for the two species along the gradient. The response of leaves to increased light was stronger for leaves of *T. cordata*, indicating higher morphological plasticity than for *F. sylvatica* (Figure 4).

## 3. Discussion

### 3.1. Mortality

#### 3.1.1. Higher Mortality outside the Fence Due to Herbivory

Mortality after the first vegetation season was marginal (Figure 1). Therefore, the seedlings’ initial survival was considered a successful start of the experiment. The low losses were potentially also due to the hydrogel treatment applied before planting which may have functioned as a mechanical protection for the fine roots [31]. The finding that more seedlings died outside the fence (Figure 1) can be explained by grazing mammals, whose browsing on seedlings commonly result in the loss of the leader shoot [32]. This loss is closely correlated to seedling mortality if approximately one third of the shoot length is browsed [33]. In the present study seedlings were sometimes browsed down to ground level or were partly missing. During the time this field experiment, grazing species such as roe deer (*Capreolus capreolus*, L. 1758), red deer (*Cervus elaphus,* L. 1758) [34], wild boar (*Sus scrofa*, L. 1758) [35], and European hares (*Lepus europaeus*, PALLAS 1778) [36] were recorded by cameras directly on the study sites. Browse damage is most intense on seedlings of 1.00–1.30 m in height [37]. The underplanted seedlings in the stands in Linde (min. size 0.16 to max. size 1.24 m) were therefore especially susceptible to grazing by mammalian herbivores.

#### 3.1.2. *F. sylvatica* Mortality Response to Light

Shade tolerance is a plant’s ability to persist in the understory at low light levels [38]. *F. sylvatica* in particular is considered to be one of, if not the most, shade tolerant species among important deciduous tree species in Germany [30,39]. *F. sylvatica* seedlings in this survey, however, responded to low light levels with the highest amount mortality in this experiment, but as light increased mortality decreased (Figure 2). This outcome is in line with other findings [40,41,42,43]. The ability of *F. sylvatica* to survive under somewhat dense *P. serotina* shrub layers with low light levels in the dense understory was expected [26,27,28]. That the mortality of *T. cordata* did not respond along the light gradient was surprising. One growing season seems to be too short to detect this effect as the seedlings are exposed to totally different biotic (e.g., competition, parasites, grazing) and abiotic factors (e.g., climate, soil, light, water, nutrients) in the forest compared to the nursery. This outcome may have been caused by other factors than the influence of light such as soil composition or soil properties, since every tree species has its own optimum in terms of soil acidity or soil aridity for instance [30,39]. Additionally, an influence by the strong competitor *P. serotina* is conceivable. Competition can arise when resources (here: light) become more available [44] so as *P. serotina* could have had reacted to the presence of the underplanted seedlings.

### 3.2. Seedling Biomass 

#### 3.2.1. No Detectable Biomass Increases after the Growing Season

The theory of plants’ optimal biomass allocation, known as the ’functional equilibrium hypotheses’ [45,46] or ’balanced growth hypothesis’ [47] suggests that plants increase allocation to organs that best access limiting resources. For example, allocation to leaves is increased when plants are limited by light [30,48]. In high light environments, plants invest more biomass to the roots [49,50] by increasing transpiration [51]. Therefore, plants react under changed light regimes with different biomass allocation strategies depending on which environmental factors change [29,48].

In this survey, the comparisons of the seedling biomass (TDM, AGB, RM) before and after the vegetation period revealed no significant changes in either trees species (Table 2). This may have been due to the destructive harvest after only one vegetation season and an acclimation process that had just started. Acclimation by seedlings to changed environmental factors has been documented in other studies [49,52,53]. Since none of our tested dry masses showed biomass increases (Table 2), the degree of acclimation of the small trees in this survey could not be clarified. Indeed, seedlings sprouts in the spring from the stock obtained from the previous year, but in our case the planting shock seemed to have strongly influenced the seedlings as they did not grow significantly during the vegetation period 2017. The seedlings’ growth performance did not reveal significant differences in terms of the mechanical treatments applied to *P. serotina*. 

However, the high survival rates of both *F. sylvatica* and *T. cordata*, in between the established population of *P. serotina* point towards the potential of underplanting. This could be a promising silvicultural alternative to outcompete the invasive species in the long run [26,27,28]. A retreatment of regrown *P. serotina* sprouts (following the initial mechanical treatments) after two to four vegetation periods would most likely strongly increase the probability of this approach to work. However, the impact of browsing should not be underestimated. If game populations are high, fencing might be necessary because native species are preferably browsed on. In addition to the expansive mechanical treatments, this would increase the costs strongly.

#### 3.2.2. Unexpected Response in *F. sylvatica* LMF

Changes in allocation depending on changed light conditions were shown in other studies (in leaves [40,49,54,55], in branches and stems [55,56], and in roots [40,49,50]).

Unexpectedly, *F. sylvatica* LMF decreased in low light conditions in this survey (Figure 3, Table 3). The effect size was minimal. This result also contradicts findings from other studies that support the theory of allocation, namely that LMF increases with shade in shade tolerant species [40,49,55]. Nevertheless, another study [41] also found an unexpected decrease in *F. sylvatica* LMF with shade. The authors there considered a fungal disease responsible for their results.

### 3.3. Shade Tolerant Species-Specific Reaction in Leaves

Results of SLA measurements in this survey confirmed findings of others that SLA decreases with increasing light availability [40,41,49,57]. The mean leaf area did not change along the light gradient, indicating that changes in SLA could be attributed to morphological changes in lamina thickness [49,58,59] and as morphological reactions of leaves to varying light conditions [29]. We found on average smaller SLA in *F. sylvatica* as compared to *T. cordata* along the entire gradient. Our model for *T. cordata* suggests a more sensitive reaction to changes in light availability in juvenile *T. cordata* as compared to juvenile *F. sylvatica* (Figure 4). Both species reacted to changed light availability resulting from mechanical treatments to *P. serotina*, which suggests an ongoing acclimation to the environmental conditions in the forest of Linde.

## 4. Materials and Methods

### 4.1. Study Area

**Figure 5:**
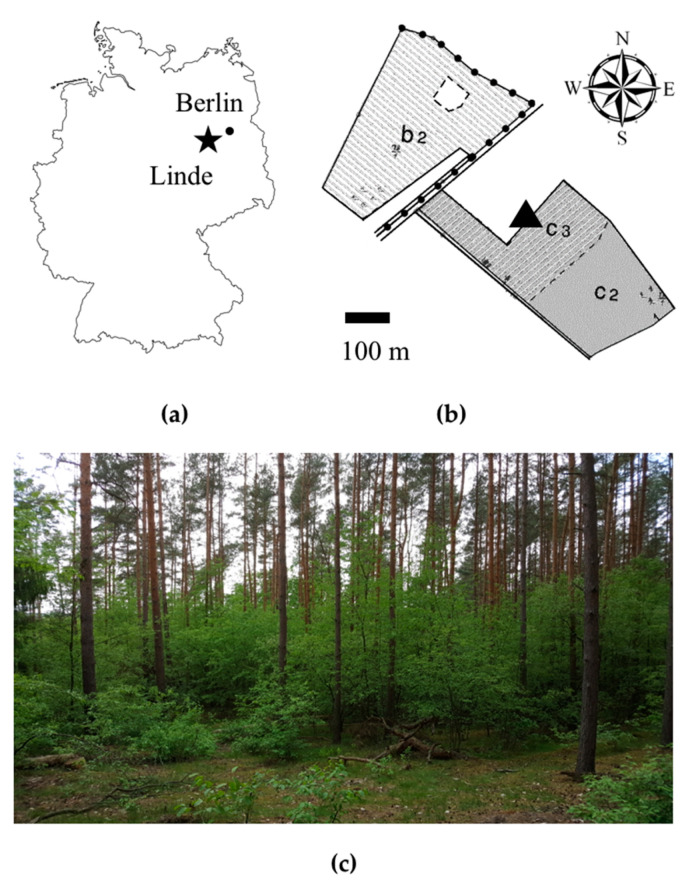
Study sites. (**a**) Location of Linde (star icon) as part of the federal state of Brandenburg. (**b**) Georeferenced forest map section with three study sites (b2, c3, c2). (**c**) Characteristic situation in the study area with established dense shrubby layers of *Prunus serotina* in the understory (date: 23.05.2017; place: stand c3, photo-location was labeled in Figure 5b).

The study area is located in Linde and belongs to the Zwillenberg-Tietz Foundation. Linde is located in eastern Germany (52°33’4.337’’ N, 12°40’55.08’’ E) inside the federal state of Brandenburg 50 km from the German capital Berlin (Figure 5a). The region around the study site is dominated by managed forests and agricultural land. Geologically characterized by flat-wave ground moraine plates and dune fields, the forest sites are rather nutrient poor. The climate of the region is classified as temperate with high temperatures in summer and moderately cold temperatures in winter. The annual average temperature is about 9 °C. Most rainfall occurs in summer during the vegetation period, with an annual precipitation of 520–572 mm [60]. The annual average temperature in 2017 was about 10 °C in the federal state of Brandenburg, which was 1.1 °C warmer, compared to the current normal periods from 1981 to 2010 (German Meteorological Service, Deutscher Wetterdienst). The precipitation in 2017 was consistently measured in 10-min intervals in a nearby meteorological station and resulted in an annual precipitation of 432.8 mm during the growing season 2017.

The study sites are at an altitude of about 63–86 m above sea level. The forests are dominated by Scots pine (*P. sylvestris* L.), but Norway spruce (*Picea abies* (L.) H.Karst.) and European larch (*Larix decidua* Mill.) also occur. Some deciduous trees, such as red oak, (*Quercus rubra* L.), small-leaved lime (*Tilia cordata* Mill.), a few individuals of chestnut (Castanea sp. Mill.), maple (Acer sp. L.), birch (Betula sp. L.), and black locust (*Robinia pseudoacacia* L.) can also be found. The non-native tree species *Prunus* *serotina* has been spreading throughout the region since the early 20th century.

The study sites were placed in relatively pure and even-aged pine stands with a *P. serotina* understory (b2, c2, c3; Figure 5b). The stands differ by thinning regime. Stand b2 was last thinned in March 2017, prior to the planting activities. Stand c3 was unthinned and stand c2 was thinned before 2006. The abundance of *P. serotina* varied slightly throughout the stands. The stand situations encountered in Linde are representative of many pine forests in eastern Germany, where *P. serotina* grows invasively (Figure 5c).

### 4.2. Study Design

**Figure 6:**
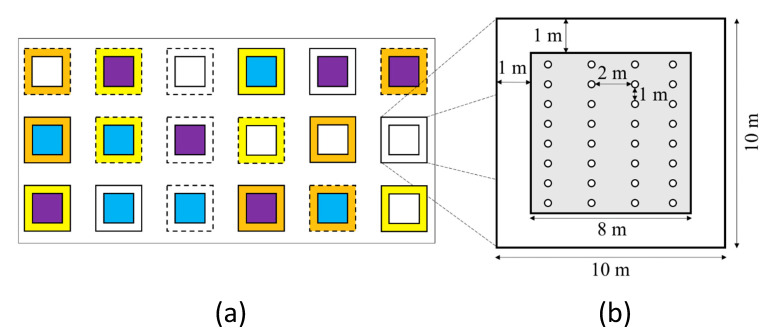
Experimental study design. (**a**) Exemplary presentation of a randomized block (*n* = 11) with 18 different plot variations. Outer square colors mark treatments applied to *P. serotina*: felling (yellow), girdling (orange), control (white); inner square colors mark underplanted tree species: *F. sylvatica* (blue), *T. cordata* (purple), no underplanting (control, white); Outer line type indicates protection: solid line (fenced), dashed line (unfenced). All plots were placed at 5 m distance from skidding trails. (**b**) Planting scheme on each 100 m^2^ plot. Fenced plots had 1 m distance between seedlings and fence. Plant spacing was 2 × 1 m, resembling a density of 5000 plant ha^−1^.

The study design was based on a block design. One block contained 18 individual 100 m^2^ plots with randomized treatments. The plots were arranged with 5 m spacing to one another and a minimum distance of 2 m to existing skidding trails. Eleven block replicates were established for a total of 197 plots (one plot was excluded), of which 90 plots were in b2, 57 in c2, and 50 in c3 (Figure 5b). The number of plots within the three stands differed due to the size of the stands. Each plot consisted of (1) a mechanical treatment applied to the *P. serotina* trees (felling or girdling), or control (without mechanical treatment), and (2) one out of two underplanted native tree species, or control (without underplanting). These plot-combinations existed fenced and unfenced. The protected plots were fenced with 1.80 m high game-proof wire to exclude grazing mammals, mainly ungulates. The plots were randomly spaced within the blocks (Figure 6a).

High shade tolerance in juvenile stages was required for the successful establishment of species under *P. serotina* [23,24,25,26,27,28]. Highly shade tolerant tree species native to Germany are *Fagus sylvatica* and *T. cordata* [30,37,39,56], therefore these two species were chosen. As planting material, 2-year-old wildstock seedlings of *F. sylvatica* and *T. cordata* were used. *F. sylvatica* seedlings had an initial biomass of 26.68 ± 11.02 g. The average biomass of *T. cordata* was 7.74 ± 4.12 g. The provenance of the planting material was Mark-Lusatia flatland for *F. sylvatica* (81005) and central and east German low and highlands for *T. cordata* (82303) [61]. Planting was conducted over 3 days in spring 2017. A total of 4189 seedlings (2109 *F. sylvatica* and 2080 *T. cordata*) were planted using a planting auger. Before the seedlings were planted, they were dipped into a hydrogel solution (GEFA Wurzelschutzgel) to improve their water storage and absorbance capabilities and to improve their overall survival rates [31]. Plant spacing was 2 × 1 m (5000 plants·ha^−1^), with 32 plants per plot (eight individuals × four rows, Figure 6b).

As mechanical treatments to control *P. serotina*, felling and girdling were applied. Before the treatments were applied to the trees, their dbh (diameter in breast height) was measured using a calliper. This study focuses on trees with a diameter in breast height ≥ 5 cm. Smaller trees (dbh < 5 cm) were counted and mechanically treated in the same way. On average, 12.53 ± 11.94 *P. serotina* trees with a dbh ≥ 5 cm grew on each plot. A total of 403 *P. serotina* trees with a dbh ≥ 5 cm were felled and removed from the plots in March and April 2017. Girdling work was done by completely removing the bark and cambium on a 15 cm wide stem circumference section beneath the lowest living branch using a chain girdler. The girdling treatment was applied in August 2017, assuming this would have the greatest detrimental effect to suppress greater sprout responses at the end of the vegetation period [62]. A total of 363 *P. serotina* trees with a dbh ≥ 5 cm were girdled.

### 4.3. Field Data Collection 

#### 4.3.1. Seedling Delivery

We received 4247 2-year-old wildstock seedlings (2139 *F. sylvatica* and 2108 *T. cordata*) from the nursery and removed 58 before planting as a basic data set (nursery plants). These seedlings (30 *F. sylvatica* and 28 *T. cordata*) were randomly chosen from the delivered plants to record the initial conditions and mass fractions for later comparisons. Overall, 4189 seedlings (2109 *F. sylvatica* and 2080 *T. cordata*) were planted of which one *F. sylvatica* and 18 *T. cordata* seedlings died directly after planting activities. Therefore, the experiment started with 4170 seedlings (2108 *F. sylvatica* and 2062 *T. cordata*).

#### 4.3.2. Seedling Mortality

The vitality of each seedling was recorded at the end of the vegetation period. Plants were considered as dead if they were either totally dried-out, had been browsed down to the ground, or were simply missing. Mortality was calculated on each plot as the ratio between dead and planted seedlings.

#### 4.3.3. Seedling Harvest

Three randomly selected seedlings were destructively harvested (above- and belowground biomass, maximum two seedlings per planting row) on each plot at the end of the growing season 2017, before the seedlings shed their leaves. This resulted in a total of 393 harvested seedlings, 198 *F. sylvatica* and 195 *T. cordata* (three harvested seedlings on 12 underplanted plots in 11 replicates minus three seedlings, because one plot was excluded).

#### 4.3.4. Light Measurements

All light measurements were carried out during the second week of July 2017. A single measurement was recorded on each plot using a hemispherical photography device equipped with a fisheye-lens (Behling, Solariscope SOL300). Measurements were conducted at 2 m above ground. In this study the ISF (indirect site factor) was used to classify the light environment on each plot. ISF is defined as proportion of diffuse radiation to open field condition, that reaches the measured point (here: per plot).

### 4.4. Lab Data Collection

Like the 58 removed nursery plants before, all 393 harvested seedlings were partitioned into leaves, branches, stems, and roots. These compartments were dried for 3 days in a temperature-controlled oven at 70 °C and weighed. Seedlings total dry mass (TDM) was the summed weight of all components: leaf mass (LM), branch mass (BM), stem mass (SM), and root mass (RM) (Equation (1)).

(1) TDM [g]=LM [g]+BM [g]+SM [g]+RM [g]

Seedling aboveground biomass (AGB) was the sum of leaf mass (LM), branch mass (BM), and stem mass (SM) (Equation (2)).

(2) AGB [g]=LM [g]+BM [g]+SM [g]

From the weight of the compartments, mass fractions could then be derived for every seedling. These were leaf mass fraction (LMF), branch mass fraction (BMF), stem mass fraction (SMF), and root mass fraction (RMF) (Equation (3)).

(3) $$\mathrm{compartment}\;\mathrm{mass}\;\mathrm{fraction}\;\left[\%\right]=\frac{\mathrm{compartment}\;\mathrm{dry}\;\mathrm{mass}\;\left[g\right]}{\mathrm{TDM}\;\left[g\right]}\times 100\;\left[\%\right]$$

Subsamples of 20 fresh leaves of *F. sylvatica* and 10 fresh leaves of *T. cordata* were randomly selected out of a collection tank after all leaves had been fully removed from each harvested seedling. The subsamples were scanned to determine mean leaf area (LA, Equation (4)) and specific leaf area (SLA, Equation (5)) for each harvested seedling. The sample size for both species differed since *T. cordata* seedlings have less leaves compared to *F. sylvatica* seedlings. The leaf area was determined using the software WinFOLIA (Regent Instruments Inc.).

(4) $$\mathrm{LA}\;\left[{\mathrm{cm}}^{2}\right]=\frac{{\mathrm{leaf}\;\mathrm{area}}_{\mathrm{subsample}}\left[{\mathrm{cm}}^{2}\right]}{{\mathrm{number}\;\mathrm{of}\;\mathrm{leaves}}_{\mathrm{subsample}}\left[n\right]}$$

(5) $$\mathrm{SLA}\;\left[{\mathrm{cm}}^{2}g^{-1}\right]=\frac{{\mathrm{leaf}\;\mathrm{area}}_{\mathrm{subsample}}\;\left[{\mathrm{cm}}^{2}\right]}{{\mathrm{leaf}\;\mathrm{dry}\;\mathrm{mass}}_{\mathrm{subsample}}\;\left[g\right]}$$

### 4.5. Data Analysis and Modelling

Statistical data analyses and graphs were processed using the statistical free software environment RStudio for R (Version 1.1.383 for Mac) [63]. Significance-level was *p* < 0.05.

Normality of distribution was checked using the Shapiro–Wilk test. Distribution was verified additionally in quantile-quantile-plots or frequency-plots. Homoscedasticity within groups (fencing, tree-species) was tested with Bartlett’s test. The non-parametric Kruskal–Wallis test (one-way ANOVA of ranks) or ANOVA were used to analyze differences in groups depending on data distribution and homoscedasticity.

Correlations between non-normal distributed data were calculated by using the non-parametric method of Kendall’s rank correlation coefficient tau (τ) to test for relationships between response variables (mortality rates, mass fractions, specific leaf area) and explanatory variables (ISF). Calculated means were compared using the independent two-sample *t*-test or Welch’s test, depending whether or not the variances for the tested groups were equal. Chi-squared test was performed to test attributes (alive or dead seedlings) for independence within groups (fencing, species).

For modelling the mass fractions along the light gradient, generalized linear models (GLM) were used with indirect site factor (ISF) as independent variable (Equation (6)), where y was the vector of the dependent variable, x the predictors matrix, α the vector of weighed predictors and ε the vector of residuals.

(6) y=x+ ε

The coefficient of determination (*R*^2^) is defined as the proportion of the variance in the dependent variable (TDM, AGB, compartment mass fractions, SLA) which can be predicted from the independent variable (light, measured as ISF) (Equation (7)). The coefficient of determination was used to evaluate the goodness of fit of the calculated GLMs.

(7) $$R^{2}=1-\frac{\mathrm{residual}\;\mathrm{sum}\;\mathrm{of}\;\mathrm{squares}}{\mathrm{total}\;\mathrm{sum}\;\mathrm{of}\;\mathrm{squares}}$$

Generalized additive models (GAM) (Equation (8)) were used to estimate mortality along the light gradient (ISF), in order to determine an unbiased detection of trends in the data [64]. Mortality was modelled as function of available light, *f* as effect-specific regression spline function of the explanatory variable (ISF), α as intercept, and ε the error term assumed to be normally distributed with a mean value of zero and with finite variance (R-package ‘mgcv’) [65].

(8) $$y=\alpha_{i}+f_{i}\left(x\right)+\;\varepsilon$$

## 5. Conclusions

Since the results of this study are based on just 1 year of observation the following conclusions need to be checked in the near future. Surprisingly, we did not find higher mortality under low light conditions in *T. cordata* than in *F. sylvatica*, which is considered to be more shade tolerant than *T. cordata*. However, we could show that *F. sylvatica* and *T. cordata* show a relatively low mortality if underplanted in Scots pine stands that had been invaded by *P. serotina.* This finding shows the potential of this approach to grant seedlings access to light by using mechanical control against *P. serotina*. Retreatments of regrown sprout might be necessary to keep up the seedling’s vitality. Fencing increases costs strongly. With one exception no changes in biomass allocation with decreasing light availability could be observed after one growing season. However, stronger morphological responses to the changed light environments as result of the treatments in both *F. sylvatica* and *T. cordata* can be expected in the up-coming years, as a result of a possibly delayed response after transplanting. The different light regimes on the plots will then most likely cause more noticeable differences in biomass allocation and growth, which will allow to identify best planting conditions. Therefore, the experiment in Linde is pursued and more data will continue to improve the recommendations that can be derived from this study for foresters dealing with the invasive tree species *P. serotina*.

## Figures and Tables

**Table 1:** Mortality per plot for *F. sylvatica* and *T. cordata* seedlings after the first growing season. Shown are detected minimum (Min), mean ± standard deviation (SD), median, and maximum (Max) mortality of dead seedlings on fenced and unfenced plots after the growing season 2017 and associated figures (Ass. figure).

Species	Fencing	Plots (*n*)	Mortality Per Plot (%)			Ass. Figure
			Min	Mean ± SD (Median)	Max	
*F. sylvatica*	Fenced	33	0	3.41 ± 4.38 (3.13)	15.63	Figure 2a
	Unfenced	33	0	5.31 ± 6.08 (3.13)	25.00	
*T. cordata*	Fenced	33	0	3.22 ± 3.54 (3.13)	12.50	Figure 2b
	Unfenced	32	0	5.87 ± 6.77 (3.13)	31.25	

**Table 2:** Seedlings’ biomass before and after the growing season 2017. Total dry mass (TDM), aboveground biomass (AGB), and belowground biomass (RM) of *F. sylvatica* and *T. cordata* before (nursery) and after the first vegetation period (harvest) in g. The leaf weights from harvested seedlings were excluded from TDM and AGB. n^1^ is the sample size of seedlings from the nursery, n^2^ is the sample size of the harvested plants. Shown are minimum (Min), mean and standard deviation (SD), median, and maximum (Max) values.

Species	Nursery					Harvest					*p* (Kruskal)
	Sample	Unit	n^1^	Min	Mean ± SD (Median)	Max	n^2^	Min	Mean ± SD (Median)	Max	
*F. sylvatica*	TDM	g	30	13.39	26.68 ± 11.02 (25.29)	59.63	98	12.42	31.33 ± 12.95 (29.16)	70.59	0.09
	AGB	g	30	5.93	12.62 ± 5.97 (11.28)	31.38	98	6.20	14.47 ± 6.34 (13.09)	35.91	0.11
	RM	g	30	6.60	14.06 ± 5.46 (13.80)	28.26	98	6.09	16.86 ± 7.21 (15.18)	39.78	0.07
*T. cordata*	TDM	g	28	3.17	7.74 ± 4.12 (7.35)	19.08	99	1.46	9.14 ± 4.84 (7.68)	24.51	0.20
	AGB	g	28	1.52	3.46 ± 1.67 (2.94)	8.65	99	0.86	3.93 ± 1.89 (3.59)	9.38	0.24
	RM	g	28	1.05	4.28 ± 2.59 (3.82)	11.27	99	0.58	5.21 ± 3.13 (4.44)	15.27	0.17

**Table 3:** Results from generalized linear models (GLM) for seedlings growth response analysis in regard to light (model: y ~ ISF). Results for *F. sylvatica* and *T. cordata* with light (ISF) as influencing factor on aboveground biomass (AGB), leaf mass fraction (LMF), branch mass fraction (BMF), stem mass fraction (SMF), root mass fraction (RMF), and specific leaf area (SLA).

Species	Response Variable	Slope	*p*-Value	Intercept	Model Equation	*R* ^2^	Ass. Figure
*F. sylvatica*	AGB	0.08	0.194	15.86	0.08 x + 15.86	0.02	
	LMF	0.04	0.016	7.94	0.04 x + 7.94	0.06	Figure 3
	BMF	−0.03	0.310	11.64	−0.03 + 11.64	0.01	
	SMF	−0.06	0.127	32.51	−0.06 + 32.51	0.02	
	RMF	0.05	0.205	47.91	0.05 + 47.91	0.02	
	SLA	−0.87	>0.001	256.14	−0.87 + 256.14	0.13	Figure 4
*T. cordata*	AGB	0.03	0.127	4.29	0.03 + 4.29	0.02	
	LMF	0.03	0.226	9.25	0.03 + 9.25	0.02	
	BMF	−0.005	0.839	3.55	−0.005 + 3.55	>0.001	
	SMF	−0.03	0.589	37.83	−0.03 + 37.83	0.003	
	RMF	0.01	0.841	49.37	0.01 + 49.37	>0.001	
	SLA	−2.01	> 0.001	343.87	−2.01 + 343.87	0.21	Figure 4

**Table 4:** Number of scanned leaves and determined specific leaf area per subsample. Number of scanned leaves and SLA in *F. sylvatica* and *T. cordata* after the vegetation season 2017. Shown are sample size (N), minimum (Min), mean and standard deviation (SD), median and maximum (Max) values.

Species	N	Scanned Leaves per Subsample (*n*)			SLA (cm^2^g ^−1^)		
		Min	Mean ± SD (Median)	Max	Min	Mean ± SD (Median)	Max
*F. sylvatica*	96	6	19.8 ± 1.8 (20)	22	170.7	237.6 ± 31.4 (232.4)	307.0
*T. cordata*	99	3	9.6 ± 1.6 (10)	13	190.6	297.7 ± 56.8 (298.0)	444.8

## Supplementary Materials

The following are available online at https://www.mdpi.com/2223-7747/9/6/677/s1, Table S1: Seedling mortality, Table S2: Seedling data, Table S3: Abbreviations, Figure 1, Figure 2, Figure 3, Figure 4, Figure 5 and Figure 6.
