# Supplementary material for: Initial Survival and Development of Planted European Beech (Fagus sylvatica L.) and Small-Leaved Lime (Tilia cordata Mill.) Seedlings Competing with Black Cherry (Prunus serotina Ehrh.)
Source: Plants (Basel). 2020 May 27;9(6):677. doi: 10.3390/plants9060677 (PMC7357064; doi:10.3390/plants9060677)
Supplement: Supplementary file 1 [file plants-09-00677-s001.zip › Table S3_Abbreviations.docx]

Abbreviations used in the manuscript "Survival and development of planted small-leaved lime (*Tilia cordata* Mill.) and European beech (*Fagus sylvatica* L.) seedlings competiting black cherry (*Prunus serotina* Ehrh.)"

| Abbreviation | Name | Definition | SI-Unit |
| --- | --- | --- | --- |
| AGB | aboveground biomass | dry mass of leaves, branches, stem | g |
| dbh | diameter in breast height | diameter in 1.3 m above ground | cm |
| BM | branch mass | branch dry mass | g |
| BMF | branch mass fraction | branch dry mass/total plant dry mass | g g^-1^ |
| GAM | generalized additive model |  |  |
| GLM | generalized linear model |  |  |
| ISF | indirect site factor | yearly diffuse sunlight availability as ratio of covered and uncovered scope |  |
| LA | mean leaf area | mean leaf area of subsamples | cm^2^ |
| LM | leaf mass | leaf dry mass | g |
| LMF | leaf mass fraction | leaf dry mass/total plant dry mass | g g^-1^ |
| RM | root mass | root dry mass | g |
| RMF | root mass fraction | root dry mass/ total plant dry mass | g g^-1^ |
| SLA | specific leaf area | ratio of leaf area to dry mass | cm^2^ g^-1^ |
| SM | stem mass | stem dry mass | g |
| SMF | stem mass fraction | stem dry mass/total plant dry mass | g g^-1^ |
| TDM | total dry mass | dry mass of (leaves), branches, stem and roots | g |
